# Supplementary material for: Sapienic Acid Metabolism Influences Membrane Plasticity and Protein Signaling in Breast Cancer Cell Lines
Source: Cells. 2022 Jan 11;11(2):225. doi: 10.3390/cells11020225 (PMC8773705; doi:10.3390/cells11020225)
Supplement: Supplementary file 1 [file cells-11-00225-s001.zip › cells-1530386-supplementary.pdf]

Supplementary Information

Sapienic acid metabolism influences membrane plasticity and protein signaling in breast cancer cell lines

Ertan Küçüksayan [1], Anna Sansone [2], Chryssostomos Chatgilialoglu [2], Tomris Ozben [3], Demet Tekeli [4], Günel Talibova [5] and Carla Ferreri [2,*]

[1] Department of Biochemistry, Faculty of Medicine, Alanya Alaaddin Keykubat University, 07070 Antalya, Turkey; ertankucuksayan@gmail.com (E.K.)
[2] Istituto per la Sintesi Organica e la Fotoreattività, Consiglio Nazionale delle Ricerche, Via Piero Gobetti 101, 40129 Bologna, Italy; anna.sansone@isof.cnr.it (A.S.); chrys@isof.cnr.it (C.C.)
[3] Department of Biochemistry, Faculty of Medicine, Akdeniz University, 07070 Antalya, Turkey; ozben@akdeniz.edu.tr (T. O.)
[4] Department of Pediatric Hematology-Oncology, Faculty of Medicine, Akdeniz University, 07070 Antalya, Turkey; dmet_cskn@hotmail.com (D.T.);
[5] Department of Histology and Embryology, Faculty of Medicine, Akdeniz University, 07070 Antalya, Turkey; guneltalibova777@gmail.com (G.T.);

* Correspondence: carla.ferreri@isof.cnr.it (C.F.)

			MCF-7		
	CTR	0.5h	1h	2h	3h
FAME[1]	(*n*=3)[2]	(*n*=3)[2]	(*n*=3)[2]	(*n*=3)[2]	(*n*=3)[2]
C14:0	1.41 ± 0.07	1.35 ± 0.15	1.61 ± 0.24	1.67 ± 0.06**	1.23 ± 0.19
C16:0	36.05 ± 1.81	36.11 ± 1.60	35.85 ± 0.64	36.83 ± 0.32	33.31 ± 2 .42
6*trans*-C16:1	0.03 ± 0.01	0.07 ± 0.04	0.06 ± 0.03	0.07 ± 0.04	0.13 ± 0.01***
6*cis*-C16:1 n-10	0.82 ± 0.37	1.36 ± 0.21	2.32 ± 0.35**	2.90 ± 0.21**	8.48 ± 1.44***
9*cis*-C16:1 n-9	2.28 ± 0.70	2.44 ± 0.15	2.26 ± 0.19	2.32 ± 0.02	2.12 ± 0.28
C18:0	32.08 ± 1.76	32.76 ± 0.74	32.07 ± 0.96	32.67 ± 0.43	29.92 ± 3.89
9*tran*s-C18:1	0.04 ± 0.01	0.09 ± 0.01**	0.04 ± 0.02	0.04 ± 0.01	0.09 ± 0.04
8*cis*-C18:1 n-10	0.22 ± 0.09	0.54 ± 0.05**	0.81 ± 0.18*	1.01 ± 0.34**	2.18 ± 0.62**
9*cis*-C18:1 n-9	13.69 ± 1.19	12.41 ± 0.76	11.92 ± 0.51	10.41 ± 0.44*	10.29 ± 2.43
11*cis*-C18:1	3.93 ± 0.47	3.78 ± 0.36	3.35 ± 0.50	3.49 ± 0.08	3.45 ± 0.62
5,8*cis*-C18:2 n-10	0.18 ± 0.04	0.21 ± 0.04	0.20 ± 0.04	0.19 ± 0.01	0.24 ± 0.02
mono-trans C18:2n-6	0.02 ± 0.02	0.05 ± 0.02	0.02 ± 0.01	0.03 ± 0.00	0.04 ± 0.03
C18:2 n-6	1.36 ± 0.36	1.16 ± 0.12	1.13 ± 0.20	1.10 ± 0.03	1.21 ± 0.37
C18:3 n-6	0.06 ± 0.02	0.11 ± 0.05	0.06 ± 0.04	0.04 ± 0.03	0.05 ± 0.02
C18:3 n-3	0.14 ± 0.04	0.16 ± 0.06	0.16 ± 0.07	0.14 ± 0.02	0.10 ± 0.02
C20:0	0.35 ± 0.01	0.36 ± 0.01	0.38 ± 0.03	0.36 ± 0.03	0.39 ± 0.02*
11*cis*-C20:1	0.40 ± 0.02	0.37 ± 0.07	0.34 ± 0.12*	0.34 ± 0.03	0.26 ± 0.06*
C20:2n-6	0.57 ± 0.14	0.50 ± 0.20	0.46 ± 0.17	0.53 ± 0.14	0.73 ± 0.24
C20:3 n-6	0.86 ± 0.30	0.93 ± 0.18	1.01 ± 0.28	0.95 ± 0.17	0.96 ± 0.23
mono-trans C20:4	0.03 ± 0.02	0.03 ± 0.01	0.20 ± 0.28	0.04 ± 0.02	0.06 ± 0.05
C20:4 n-6	2.08 ± 0.80	2.27 ± 0.31	1.36 ± 1.1 9	2.11 ± 0.08	2.19 ± 0.38
C20:5 n-3	0.40 ± 0.17	0.46 ± 0.06	0.42 ± 0.10	0.42 ± 0.04	0.38 ± 0.04
C22:5 n-3	1.25 ± 0.34	0.99 ± 0.02	0.93 ± 0.13	0.94 ± 0.06	0.99 ± 0.15
C22:6 n-3	1.63 ± 0.32	1.43 ± 0.16	1.41 ± 0.20	1.36 ± 0.12	1.31 ± 0.27
SFA	69.89 ± 3.51	70.57 ± 2.01	69.92 ± 1.68	71.53 ± 0.26	64.85 ± 6.49
MUFA	17.45 ± 1.39	17.21 ± 0.71	17.69 ± 1.10	17.03 ± 0.41	23.42 ± 4.80
PUFA	8.52 ± 1.43	8.22 ± 1.04	7.14 ± 1.58	7.78 ± 0.34	8.16 ± 1.23
n-6	4.92 ± 1.04	4.98 ± 0.74	4.02 ± 1.30	4.73 ± 0.24	5.13 ± 0.80
n-3	3.43 ± 0.60	3.04 ± 0.29	2.92 ± 0.43	2.87 ± 0.13	2.78 ± 0.44
n-6/n-3	1.45 ± 0.29	1.63 ± 0.11	1.38 ± 0.38	1.65 ± 0.06	1.84 ± 0.00
n-10	1.22 ± 0.43	2.11 ± 0.21*	3.33 ± 0.56**	4.10 ± 0.45**	10.91 ± 2.01**
total *trans*	0.13 ± 0.04	0.24 ± 0.02	0.32 ± 0.28	0.18 ± 0.04	0.32 ± 0.08*
sapienic/palmitoleic	0.37 ± 0.14	0.56 ± 0.10	1.03 ± 0.16	1.25 ± 0.08***	4.00 ± 0.3***

TABLE S1. Fatty acid residues of phospholipids isolated from MCF-7 cells in control cultures experiments and in cultures where 50 μM sapienic acid was added, isolated after the indicated times. [1] FAME (fatty acid methyl esters) are expressed as relative quantitative percentages (% rel. quant.) calculated from the quantities of fatty acids obtained by GC analysis using calibration with standard references. The recognition is made by standard references and the recognized peaks are >97% of total GC peaks. The statistics was carried out in comparison with controls (CTR) grown under the same conditions without fatty acid; * p value ≤0.04; **p value≤0.007; ***p value ≤0.0009.

	MDA-MB-231				
	CTR	0.5h	1h	2h	3h
FAME[1]	(*n*=3)[2]	(*n*=3)[2]	(*n*=3)[2]	(*n*=3)[2]	(*n*=3)[2]
C14:0	1.29 ± 0.42	1.18 ± 0.23	1.35 ± 0.36	1.04 ± 0.08	0.81 ± 0.19
C16:0	27.16 ± 1.17	22.35 ± 0.68**	19.68 ± 0.60***	18.76 ± 0.25***	25.97 ± 5.40
6*trans*-C16:1	0.15 ± 0.02	0.13 ± 0.07	0.18 ± 0.04	0.18 ± 0.04	0.17 ± 0.06
6*cis*-C16:1 n-10	2.38 ± 0.21	6.26 ± 0.53***	6.11 ± 1.30**	6.34 ± 1.16**	6.97 ± 0.47***
9*cis*-C16:1 n-9	1.00 ± 0.06	0.90 ± 0.18	0.74 ± 0.24	0.69 ± 0.06**	0.93 ± 0.09
C18:0	16.21 ± 0.99	12.94 ± 0.76*	13.43 ± 0.98*	13.35 ± 1.48*	11.90 ± 0.29**
9*trans*-C18:1	0.07 ± 0.03	0.10 ± 0.01	0.07 ± 0.02	0.08 ± 0.00	0.06 ± 0.03
8*cis*-C18:1 n-10	0.83 ± 0.10	1.45 ± 0.38	2.02 ± 0.97	2.37 ± 1.03	2.24 ± 0.25***
9*cis*-C18:1 n-9	16.34 ± 0.54	30.40 ± 2.22	29.92 ± 1.17***	31.24 ± 1.41***	20.35 ± 0.36***
11*cis*-C18:1	5.58 ± 0.39	3.82 ± 0.18	3.62 ± 0.93*	3.56 ± 0.60**	4.88 ± 0.74
5,8*cis*-C18:2 n-10	0.47 ± 0.06	0.57 ± 0.20	0.57 ± 0.12	0.44 ± 0.06	0.45 ± 0.06
mono-trans C18:2n-6	0.09 ± 0.04	0.06 ± 0.02	0.05 ± 0.04	0.09 ± 0.08	0.09 ± 0.03
C18:2 n-6	5.12 ± 0.14	6.65 ± 0.07***	7.33 ± 0.23***	7.28 ± 0.24***	4.80 ± 0.70
C18:3 n-6	0.19 ± 0.02	0.25 ± 0.2	0.13 ± 0.02*	0.16 ± 0.04	0.22 ± 0.08
C18:3 n-3	0.19 ± 0.04	0.16 ± 0.06	0.13 ± 0.06*	0.15 ± 0.06*	0.27 ± 0.02*
C20:0	0.55 ± 0.17	0.43 ± 0.08	0.45 ± 0.08*	0.37 ± 0.19**	0.42 ± 0.17
11*cis*-C20:1	0.43 ± 0.07	0.24 ± 0.04*	0.24 ± 0.04*	0.25 ± 0.02*	0.36 ± 0.11
C20:2n-6	0.92 ± 0.20	0.65 ± 0.19	0.79 ± 0.13	0.97 ± 0.30	1.03 ± 0.34
C20:3 n-6	4.02 ± 0.22	1.71 ± 0.23***	2.44 ± 0.66*	2.41 ± 0.46**	3.59 ± 0.67
mono-trans C20:4	0.19 ± 0.09	0.06 ± 0.03	0.10 ± 0.04	0.08 ± 0.06	0.09 ± 0.03
C20:4 n-6	11.23 ± 0.68	6.81 ± 0.80**	7.11 ± 1.43*	7.29 ± 1.03**	8.56 ± 1.02*
C20:5 n-3	0.18 ± 0.11	0.46 ± 0.29	0.14 ± 0.06	0.12 ± 0.04	0.14 ± 0.02
C22:5 n-3	2.61 ± 0.54	1.28 ± 0.63*	1.47 ± 0.40*	1.49 ± 0.30*	2.62 ± 1.00
C22:6 n-3	2.55 ± 0.58	1.73 ± 0.67	1.85 ± 0.10	1.55 ± 0.21*	2.76 ± 0.90
SFA	45.21 ± 2.30	36.91 ± 0.52**	34.92 ± 0.62**	33.52 ± 1.27**	39.09 ± 5.07
MUFA	21.06 ± 0.58	39.39 ± 2.40***	39.09 ± 3.00***	40.97 ± 3.24***	30.90 ± 0.21***
PUFA	27.47 ± 2.12	20.27 ± 2.00*	21.97 ± 2.14*	21.86 ± 1.91*	24.43 ± 4.10
n-6	21.48 ± 0.91	16.07 ± 1.05**	17.81 ± 1.86*	18.12 ± 1.51*	18.20 ± 2.42
n-3	5.53 ± 1.17	3.62 ± 1.03	3.59 ± 0.39	3.30 ± 0.54*	5.78 ± 1.87
n-6/n-3	3.98 ± 0.64	4.63 ± 1.06	4.96 ± 0.05	5.54 ± 0.65*	3.30 ± 0.73
n-10	3.69 ± 0.16	8.28 ± 0.56***	8.69 ± 2.38*	9.14 ± 2.17*	9.66 ± 0.43***
total *trans*	0.50 ± 0.10	0.35 ± 0.10	0.40 ± 0.09	0.43 ± 0.04	0.41 ± 0.05
sapienic/palmitoleic	2.37 ± 0.09	7.20 ± 1.93*	9.24 ± 5.07	9.30 ± 2.28	7.60 ± 1.22**

TABLE S2. Fatty acid residues of phospholipids isolated from MDA-MB-231 cells in control cultures experiments and in cultures where 50 µM sapienic acid was added, isolated after the indicated times. [1] FAME (fatty acid methyl esters) are expressed as relative quantitative percentages (% rel. quant.) calculated from the quantities of fatty acids obtained by GC analysis using calibration with standard references. The recognition is made by standard references and the recognized peaks are >97% of total GC peaks. The statistics was carried out in comparison with controls (CTR) grown under the same conditions without fatty acid; * p value ≤0.04; **p value≤0.007; ***p value ≤0.0009.

	BT-20				
	CTR	0.5h	1h	2h	3h
FAME[1]	(*n*=3)[2]	(*n*=3)[2]	(*n*=3)[2]	(*n*=3)[2]	(*n*=3)[2]
C14:0	1.32 ± 0.21	0.84 ± 0.07*	2.34 ± 0.95	3.36 ± 0.66**	1.76 ± 0.32
C16:0	29.22 ± 0.67	26.08 ± 1.26*	24.35 ± 4.33	23.69 ± 1.38**	23.11 ± 0.89***
6*trans*-C16:1	0.18 ± 0.03	0.17 ± 0.05	0.13 ± 0.03	0.140 ± .001	0.28 ± 0.06*
6*cis*-C16:1 n-10	2.86 ± 0.27	3.01 ± 0.88	3.58 ± 0.92	6.35 ± 0.51***	12.91 ± 2.21**
9*cis*-C16:1 n-9	1.03 ± 0.09	0.99 ± 0.09	1.47 ± 0.75	1.07 ± 0.07	1.06 ± 0.61
C18:0	13.76 ± 0.33	13.11 ± 0.74	12.85 ± 2.84	12.98 ± 0.68	12.85 ± 0.74
9*trans*-C18:1	0.09 ± 0.05	0.11 ± 0.03	0.28 ± 0.06**	0.19 ± 0.04	0.08 ± 0.04
8*cis*-C18:1 n-10	0.67 ± 0.04	1.09 ± 0.30	1.21 ± 0.50	1.28 ± 0.08***	1.15 ± 0.33
9*cis*-C18:1 n-9	18.78 ± 0.60	18.70 ± 0.41	18.12 ± 0.89	16.93 ± 0.49*	16.14 ± 1.30*
11*cis*-C18:1	3.45 ± 0.19	3.36 ± 0.25	3.34 ± 0.47	3.14 ± 0.24	2.64 ± 0.12**
5,8*cis*-C18:2 n-10	0.25 ± 0.03	0.36 ± 0.02**	0.37 ± 0.18	0.33 ± 0.09	0.35 ± 0.11
mono-trans C18:2n-6	0.04 ± 0.02	0.10 ± 0.07	0.04 ± 0.02	0.14 ± 0.03**	0.07 ± 0.05
C18:2 n-6	4.91 ± 0.07	5.02 ± 0.13	5.46 ± 0.18**	5.42 ± 0.21*	4.83 ± 0.25
C18:3 n-6	0.19 ± 0.04	0.31 ± 0.10	0.33 ± 0.15	0.26 ± 0.14	0.20 ± 0.02
C18:3 n-3	0.08 ± 0.00	0.21 ± 0.05	0.41 ± 0.19*	0.20 ± 0.11	0.26 ± 0.06**
C20:0	0.50 ± 0.02	0.52 ± 0.01	0.73 ± 0.51	0.46 ± 0.06	0.42 ± 0.24
11*cis*-C20:1	0.26 ± 0.02	0.44 ± 0.21	0.97 ± 0.35*	0.54 ± 0.28	0.25 ± 0.09
C20:2n-6	0.58 ± 0.10	0.54 ± 0.13	0.70 ± 0.16	2.17 ± 0.84*	0.57 ± 0.37
C20:3 n-6	5.29 ± 0.22	6.55 ± 0.43*	5.18 ± 0.06	5.42 ± 0.61	5.75 ± 0.22
mono-trans C20:4	0.04 ± 0.01	0.09 ± 0.01**	0.23 ± 0.13*	0.08 ± 0.02*	0.05 ± 0.04
C20:4 n-6	9.17 ± 0.27	10.45 ± 0.38**	8.99 ± 0.85	9.57 ± 1.34*	9.08 ± 0.39
C20:5 n-3	0.41 ± 0.01	0.48 ± 0.12	0.9 ± 0.02***	0.69 ± 0.13*	0.66 ± 0.11*
C22:5 n-3	3.47 ± 0.07	4.09 ± 0.29*	3.36 ± 0.47	3.10 ± 0.17*	3.29 ± 0.45
C22:6 n-3	2.59 ± 0.24	3.15 ± 0.48	2.98 ± 0.68	2.37 ± 0.21	2.78 ± 0.36
SFA	44.79 ± 0.50	40.55 ± 1.84*	40.28 ± 2.84	40.49 ± 1.39**	38.13 ± 0.87***
MUFA	27.05 ± 0.10	27.25 ± 1.75	28.69 ± 1.14	29.32 ± 0.76**	34.16 ± 0.96***
PUFA	26.95 ± 0.67	31.15 ± 0.28***	28.76 ± 2.56	29.54 ± 0.95*	27.78 ± 2.17
n-6	20.14 ± 0.47	22.87 ± 0.83**	20.66 ± 1.30	22.84 ± 0.89*	20.43 ± 1.19
n-3	6.56 ± 0.24	7.93 ± 0.87	7.74 ± 1.18	6.37 ± 0.35	7.00 ± 0.91
n-6/n-3	3.07 ± 0.06	2.91 ± 0.43	2.70 ± 0.27	3.60 ± 0.25*	2.94 ± 0.23
n-10	3.78 ± 0.33	4.46 ± 1.13	5.15 ± 1.09	7.97 ± 0.60***	14.41 ± 1.82***
total *trans*	0.36 ± 0.06	0.46 ± 0.14	0.68 ± 0.21**	0.55 ± 0.03*	0.49 ± 0.15
sapienic/palmitoleic	2.77 ± 0.05	3.02 ± 0.69	2.69 ± 0.80	5.96 ± 0.73**	14.49 ± 6.42*

TABLE S3. Fatty acid residues of phospholipids isolated from BT-20 cells in control cultures experiments and in cultures where 50 μM sapienic acid was added, isolated after the indicated times. [1] FAME (fatty acid methyl esters) are expressed as relative quantitative percentages (% rel. quant.) calculated from the quantities of fatty acids obtained by GC analysis using calibration with standard references. The recognition is made by standard references and the recognized peaks are >97% of total GC peaks. The statistics was carried out in comparison with controls (CTR) grown under the same conditions without fatty acid; * p value ≤0.04; **p value≤0.007; ***p value ≤0.0006.

FAME[1]	MCF-7 CTR (*n*=3)[2]	CTR-PC[3] (*n*=3)[2]
C14:0	1.41 ± 0.07	1.27 ± 0.18
C16:0	36.05 ± 1.81	35.34 ± 4.10
6*trans*-C16:1	0.03 ± 0.01	0.04 ± 0.01
6*cis*-C16:1 n-10	0.82 ± 0.37	0.88 ± 0.16
9*cis*-C16:1 n-9	2.28 ± 0.70	2.61 ± 0.71
C18:0	32.08 ± 1.76	31.87 ± 3.89
9*tran*s-C18:1	0.04 ± 0.01	0.08 ± 0.04
8*cis*-C18:1 n-10	0.22 ± 0.09	0.21 ± 0.06
9*cis*-C18:1 n-9	13.69 ± 1.19	13.74 ± 3.78
11*cis*-C18:1	3.93 ± 0.47	3.86 ± 0.91
5,8*cis*-C18:2 n-10	0.18 ± 0.04	0.16 ± 0.05
mono-trans C18:2n-6	0.02 ± 0.02	0.05 ± 0.03
C18:2 n-6	1.36 ± 0.36	1.21 ± 0.31
C18:3 n-6	0.06 ± 0.02	0.08 ± 0.03
C18:3 n-3	0.14 ± 0.04	0.14 ± 0.01
C20:0	0.35 ± 0.01	0.30 ± 0.04
11*cis*-C20:1	0.40 ± 0.02	0.38 ± 0.09
C20:2n-6	0.57 ± 0.14	0.66 ± 0.24
C20:3 n-6	0.86 ± 0.30	1.06 ± 0.29
mono-trans C20:4	0.03 ± 0.02	0.02 ± 0.01
C20:4 n-6	2.08 ± 0.80	2.80 ± 0.64
C20:5 n-3	0.40 ± 0.17	0.58 ± 0.24
C22:5 n-3	1.25 ± 0.34	1.05 ± 0.31
C22:6 n-3	1.63 ± 0.32	1.35 ± 0.49
SFA	69.89 ± 3.51	68.79 ± 7.94
MUFA	17.45 ± 1.39	17.89 ± 4.64
PUFA	8.52 ± 1.43	9.10 ± 2.40
n-6	4.92 ± 1.04	5.81 ± 1.43
n-3	3.43 ± 0.60	3.13 ± 1.04
n-6/n-3	1.45 ± 0.29	1.91 ± 0.36
n-10	1.22 ± 0.43	1.25 ± 0.08
total *trans*	0.13 ± 0.04	0.19 ± 0.07
sapienic/palmitoleic	0.37 ± 0.14	0.35 ± 0.09

TABLE S4. Fatty acid residues of phospholipids isolated from MCF-7 cells in control cultures experiments and in cultures where 50 μM sapienic acid was added and washed after 2 minutes (CTR-PC, pulse and chase experiment). [1] FAME (fatty acid methyl esters) are expressed as relative quantitative percentages (% rel. quant.) calculated from the quantities of fatty acids obtained by GC analysis using calibration with standard references. The recognition is made by standard references and the recognized peaks are >97% of total GC peaks. [2] The values are expressed as mean ± sd (standard deviation) of the experiments performed in triplicates.

FAME[1]	MDA-MB-231 CTR (n=3)[2]	CTR-PC[3] (n=3)[2]
C14:0	1.29 ± 0.42	2.05 ± 0.37
C16:0	27.16 ± 1.17	30.87 ± 3.40
6*trans*-C16:1	0.15 ± 0.02	0.15 ± 0.02
6*cis*-C16:1 n-10	2.38 ± 0.21	5.99 ± 0.62***
9*cis*-C16:1 n-9	1.00 ± 0.06	0.97 ± 0.05
C18:0	16.21 ± 0.99	14.51 ± 0.56
9*trans*-C18:1	0.07 ± 0.03	0.09 ± 0.01
8*cis*-C18:1 n-10	0.83 ± 0.10	1.70 ± 0.20**
9*cis*-C18:1 n-9	16.34 ± 0.54	15.35 ± 0.69
11*cis*-C18:1	5.58 ± 0.39	4.75 ± 0.58
5,8*cis*-C18:2 n-10	0.47 ± 0.06	0.58 ± 0.02*
mono-trans C18:2n-6	0.09 ± 0.04	0.09 ± 0.03
C18:2 n-6	5.12 ± 0.14	5.04 ± 0.28
C18:3 n-6	0.19 ± 0.02	0.19 ± 0.04
C18:3 n-3	0.19 ± 0.04	0.19 ± 0.06
C20:0	0.55 ± 0.17	0.54 ± 0.02
11*cis*-C20:1	0.43 ± 0.07	0.38 ± 0.16
C20:2n-6	0.92 ± 0.20	0.83 ± 0.06
C20:3 n-6	4.02 ± 0.22	3.03 ± 0.57*
mono-trans C20:4	0.19 ± 0.09	0.18 ± 0.02
C20:4 n-6	11.23 ± 0.68	7.73 ± 1.62*
C20:5 n-3	0.18 ± 0.11	0.21 ± 0.03
C22:5 n-3	2.61 ± 0.54	1.70 ± 0.64
C22:6 n-3	2.55 ± 0.58	2.07 ± 0.87
SFA	45.21 ± 2.30	47.97 ± 3.30
MUFA	21.06 ± 0.58	24.49 ± 1.00
PUFA	27.47 ± 2.12	21.57 ± 3.98
n-6	21.48 ± 0.91	16.83 ± 2.46*
n-3	5.53 ± 1.17	4.16 ± 1.53
n-6/n-3	3.98 ± 0.64	4.31 ± 1.16
n-10	3.69 ± 0.16	8.27 ± 0.64***
total *trans*	0.50 ± 0.10	0.51 ± 0.02
sapienic/palmitoleic	2.37 ± 0.09	6.17 ± 0.73***

TABLE S5. Fatty acid residues of phospholipids isolated from MDA-MB-231 cells in control cultures experiments and in cultures where 50 μM sapienic acid was added and washed after 2 minutes (CTR-PC, pulse and chase experiment). [1] FAME (fatty acid methyl esters) are expressed as relative quantitative percentages (% rel. quant.) calculated from the quantities of fatty acids obtained by GC analysis using calibration with standard references. The recognition is made by standard references and the recognized peaks are >97% of total GC peaks. [2] The values are expressed as mean ± sd (standard deviation) of the experiments performed in triplicates. * p value ≤0.04; **p value≤0.006; ***p value ≤0.0009.

	BT-20	
FAME[1]	CTR (n=3)[2]	CTR-PC[3] (n=3)[2]
C14:0	1.32 ± 0.21	1.15 ± 0.21
C16:0	29.22 ± 0.67	28.14 ± 1.91
6*trans*-C16:1	0.18 ± 0.03	0.16 ± 0.05
6*cis*-C16:1 n-10	2.86 ± 0.27	3.14 ± 0.83
9*cis*-C16:1 n-9	1.03 ± 0.09	0.94 ± 0.01
C18:0	13.76 ± 0.33	12.92 ± 2.71
9*tran*s-C18:1	0.09 ± 0.05	0.10 ± 0.41
8*cis*-C18:1 n-10	0.67 ± 0.04	0.95 ± 0.45
9*cis*-C18:1 n-9	18.78 ± 0.60	20.60 ± 2.93
11*cis*-C18:1	3.45 ± 0.19	3.29 ± 0.56
5,8*cis*-C18:2 n-10	0.25 ± 0.03	0.21 ± 0.03
mono-trans C18:2n-6	0.04 ± 0.02	0.05 ± 0.02
C18:2 n-6	4.91 ± 0.07	5.12 ± 0.19
C18:3 n-6	0.19 ± 0.04	0.13 ± 0.05
C18:3 n-3	0.08 ± 0.00	0.10 ± 0.02*
C20:0	0.50 ± 0.02	0.42 ± 0.04*
11*cis*-C20:1	0.26 ± 0.02	0.32 ± 0.07
C20:2n-6	0.58 ± 0.10	0.67 ± 0.24
C20:3 n-6	5.29 ± 0.22	6.06 ± 0.07**
mono-trans C20:4	0.04 ± 0.01	0.06 ± 0.03
C20:4 n-6	9.17 ± 0.27	10.43 ± 0.49*
C20:5 n-3	0.41 ± 0.01	0.60 ± 0.02***
C22:5 n-3	3.47 ± 0.07	3.86 ± 0.22*
C22:6 n-3	2.59 ± 0.24	2.06 ± 0.90
SFA	44.79 ± 0.50	42.63 ± 0.67*
MUFA	27.05 ± 0.10	29.23 ± 3.70
PUFA	26.95 ± 0.67	29.24 ± 0.47**
n-6	20.14 ± 0.47	22.40 ± 0.50**
n-3	6.56 ± 0.24	6.63 ± 0.70
n-6/n-3	3.07 ± 0.06	3.41 ± 0.45
n-10	3.78 ± 0.33	4.29 ± 1.27
total *trans*	0.36 ± 0.06	0.30 ± 0.45
sapienic/palmitoleic	2.77 ± 0.05	3.35 ± 0.88

TABLE S6. Fatty acid residues of phospholipids isolated from BT-20 cells in control cultures experiments and in cultures where 50 μM sapienic acid was added and washed after 2 minutes (CTR-PC, pulse and chase experiment). [1] FAME (fatty acid methyl esters) are expressed as relative quantitative percentages (% rel. quant.) calculated from the quantities of fatty acids obtained by GC analysis using calibration with standard references. The recognition is made by standard references and the recognized peaks are >97% of total GC peaks. [2] The values are expressed as mean ± sd (standard deviation) of the experiments performed in triplicates. * p value ≤0.04; **p value≤0.009; ***p value ≤0.0006.

HPLC detection of lipid classes

Using the HPLC conditions described in the main text, we first injected the following standard references for the main lipid classes present in the cell membrane pellets:

POPC (1-palmitoyl-2-oleoyl-sn- glycero-3-phosphocholine)

POPE (1-palmitoyl-2-oleoyl-sn-glycero-3-phosphoethanolamine)

SM (sphingomyelin)

CHO (cholesterol)

POPS (1-palmitoyl-2-oleoyl-sn-glycero-3-phosphoserine).

We prepared stock solutions of 1mg/mL in 1 M ammonium formate/MeOH/2-propanol:10/20/70) and operated dilutions to perform multiple points of calibration curves (at least 6 points), determining the instrumental parameters: LOQ (limit of quantitation) LOD (limit of detection) and the linearity of calibration curves R^2.

LOQ, LOD and R^2 of LIPID STANDARDS

STANDARD REFERENCES	LOQ ng/mL	LOD ng/mL	R^2
POPC	781.2	260.4	0.9999
POPE	390.6	134.6	0.9985
SM	975.5	314.67	0.9999
CHO	0.244	0.069	0.9997
POPS	625.2	312.5	0.9892

The repeatability of the standards was expressed as Relative Standard Deviation (RSD (%) =(SD/mean) ×100) of the replicates (n=3) and calculated for each peak area and retention time (r.t.). RSD% values are related to the following concentrations of the standards: 0.5 mg/mL and 0.125 mg/mL.

REPEATABILITY of the STANDARDS

STANDARD REFERENCES	RDS% (0.5 mg/mL) peak area	RDS% (0.5 mg/mL) r.t.	RDS% (0.125 mg/mL) peak area	RDS% (0.125mg/mL) r.t.
POPC	0.37	0.32	1.2	0.31
POPE	1.84	0.71	1.43	0.72
SM	0.57	0.73	0.84	0.74
CHO	0.43	0.39	0.25	0.41
POPS	0.44	0.81	0.71	0.72

INTER-DAY REPEATABILITY of the STANDARDS

The inter-day repeatability of the standards, monitored in two consecutive days, is expressed as Relative Standard Deviation (RSD (%) = (SD/mean) ×100) of the total replicates (n=6, n=3/day) and calculated for each peak area and retention time. RSD% values related to the concentration of the standards of 0.25 mg/mL and 0.0625 mg/mL

STANDARD REFERENCES	RDS% (0.250 mg/mL) PEAK AREA	RDS% (0.250 mg/mL) R.T.	RDS% (0.0625 mg/mL) PEAK AREA	RDS% (0.0625mg/mL) R.T
POPC	0.69	0.34	1.47	0.37
POPE	1.17	0.69	1.3	0.41
SM	1.61	0.69	1.53	0.53
CHO	0.2	0.40	0.99	0.43
POPS	1.62	0.75	1.05	0.72

**MCF-7**				
Lipids	CTR (*n*=3)	1h (*n*=3)	2h (*n*=3)	3h (*n*=3)
PS	31.31 ± 4.63	35.28 ± 1.97	35.94 ± 2.14	26.72 ± 4.88
CHO	6.30 ± 1.97	2.84 ± 0.96	3.35 ± 0.56	3.45 ± 1.37
PE	16.32 ± 5.27	11.40 ± 4.77	11.37 ± 8.44	22.20 ± 7.13
SM	3.64 ± 1.02	6.38 ± 1.42	1.45 ± 0.33*	5.88 ± 2.95
PC	42.42 ± 4.64	44.10 ± 4.96	47.89 ± 6.17	41.75 ± 1.43

Table S7. Main lipid classes isolated from MCF-7 cells treated with 50 μM sapienic acid at the indicated times. Values are expressed as relative quantitative percentages (% rel. quant.) calculated from the quantities of lipids obtained by HPLC analysis identified and calibrated by the standard references as described in Materials and Methods. Values are mean ± sd (deviation of standard) of experiments performed in triplicates. The statistics was carried out in comparison with controls (CTR) grown under the same conditions without fatty acid; * p value ≤0.03; PS = phosphatidyl serine, CHO = cholesterol; PE = phosphatidyl ethanolamine; SM = sphingomyelin; PC = phosphatidyl choline.

MDA-MB-231				
Lipids	CTR (*n*=3)	1h (*n*=3)	2h (*n*=3)	3h (*n*=3)
PS	32.02 ± 6.19	33.77 ± 1.46	39.21 ± 4.98	35.01 ± 1.66
CHO	1.98 ± 0.43	2.15 ± 0.44	3.85 ± 1.20	1.14 ± 0.10*
PE	18.78 ± 3.80	15.53 ± 1.31	18.74 ± 2.44	20.31 ± 3.18
SM	2.20 ± 0.76	3.31 ± 0.82	3.49 ± 1.00	3.75 ± 0.74
PC	45.02 ± 3.47	45.24 ± 1.52	34.71 ± 4.20*	39.80 ± 3.65

Table S8. Main lipid classes isolated from MDA-MB-231 cells treated with 50 μM sapienic acid at the indicated times. Values are expressed as relative quantitative percentages (% rel. quant.) calculated from the quantities of lipids obtained by HPLC analysis identified and calibrated by the standard references as described in Materials and Methods. Values are mean ± sd (deviation of standard) of experiments performed in triplicates. The statistics was carried out in comparison with controls (CTR) grown under the same conditions without fatty acid; * p value ≤0.03; PS = phosphatidyl serine, CHO = cholesterol; PE = phosphatidyl ethanolamine; SM = sphingomyelin; PC = phosphatidyl choline.

BT-20				
Lipids	CTR (*n*=3)	1h (*n*=3)	2h (*n*=3)	3h (*n*=3)
PS	24.22 ± 2.97	23.48 ± 0.80	26.12 ± 5.54	24.48 ± 2.73
CHO	3.85 ± 0.98	2.37 ± 0.34	2.80 ± 0.79	6.08 ± 2.85
PE	23.46 ± 5.83	26.92 ± 1.41	25.20 ± 3.70	16.85 ± 3.71
SM	5.28 ± 0.40	6.03 ± 0.08*	6.88 ± 1.57	6.38 ± 0.04**
PC	43.18 ± 1.78	41.20 ± 0.72	39.00 ± 5.64	46.20 ± 3.69

Table S9. Main lipid classes isolated from BT-20 cells treated with 50 μM sapienic acid at the indicated times. Values are expressed as relative quantitative percentages (% rel. quant.) calculated from the quantities of lipids obtained by HPLC analysis identified and calibrated by the standard references as described in Materials and Methods. Values are mean ± sd (deviation of standard) of experiments performed in triplicates. The statistics was carried out in comparison with controls (CTR) grown under the same conditions without fatty acid; * p value ≤0.03; * *p value ≤0.004. PS = phosphatidyl serine, CHO = cholesterol; PE = phosphatidyl ethanolamine; SM = sphingomyelin; PC = phosphatidyl choline.

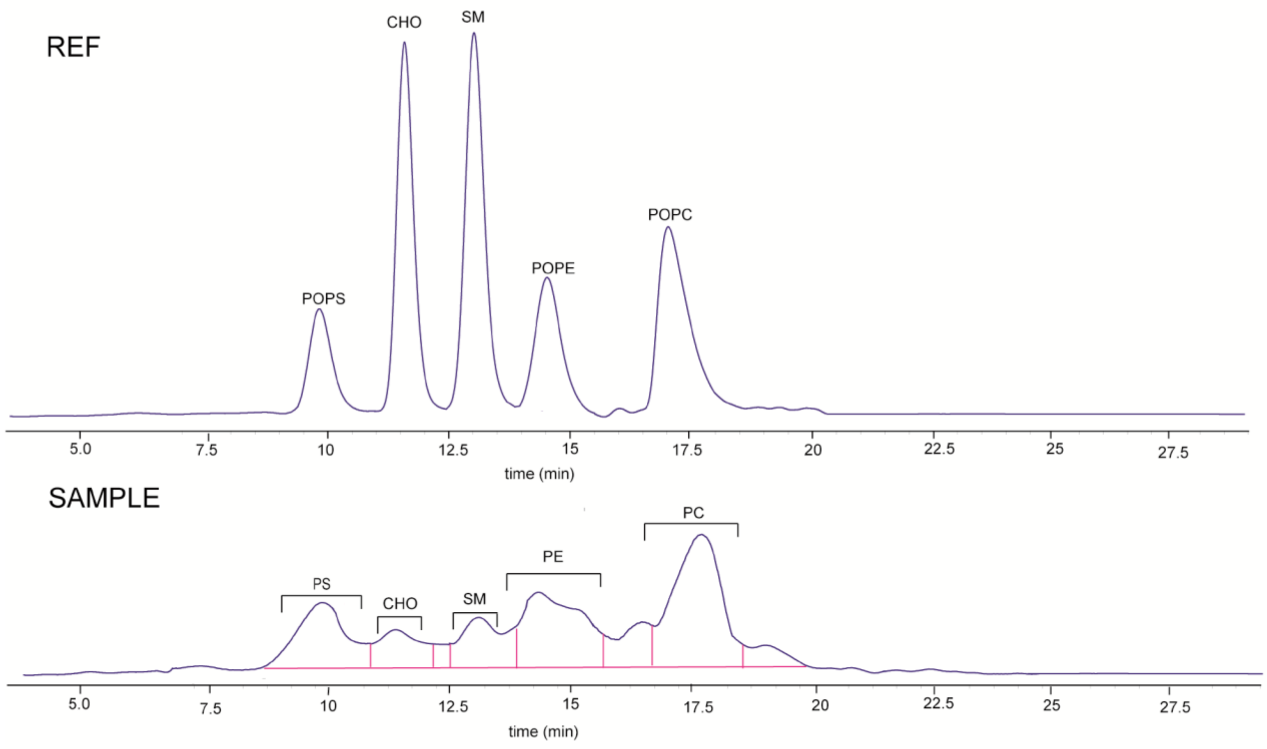

Figure S1. Representative HPLC runs of the standard references and one sample of the membrane pellet, eluted under the conditions indicated in the main text.

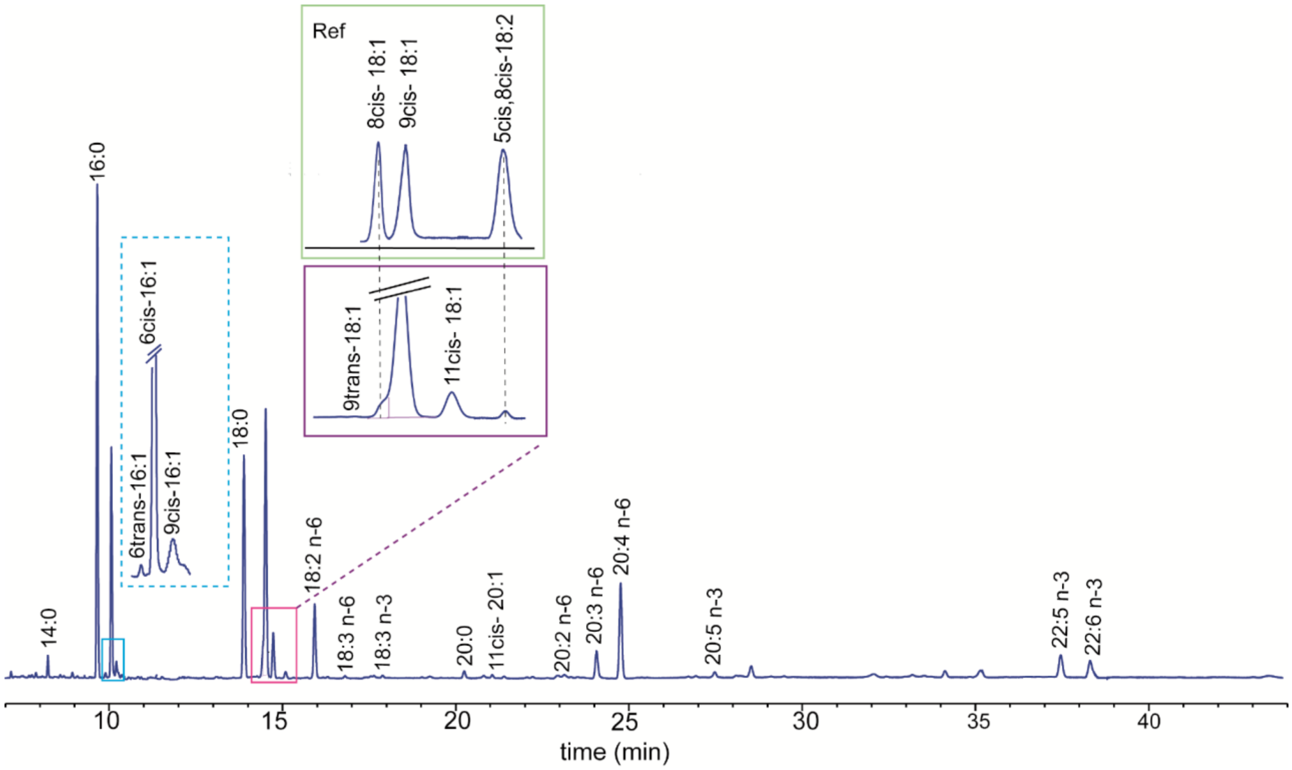

Figure S2. Representative GC analysis of the fatty acid methyl esters (FAME) obtained from membrane phospholipid isolation and work-up as described in the main text. In the inset the separation and identification of the n-10 fatty acids (6cis-16:1, 8cis-18:1 and 5cis,8cis-18:2) using commercially available standard references are shown. The quantification of the main fatty acid peaks was obtained by calibrated procedures and reported in Tables and Figures.

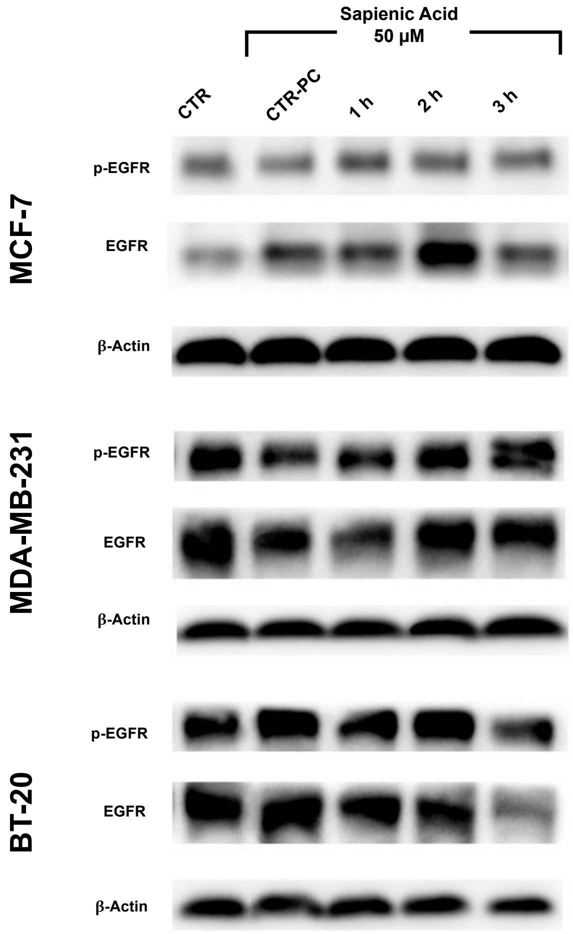

Figure S3. Levels of p-EGFR and EGFR proteins (Western blot) in whole-cell lysates isolated from the breast cancer cell lines, using β-actin as loading controls. 30 μg of protein were loaded for both whole-cell lysate. Detections carried out at 0 (CTR), 1, 2, 3 hours of incubation at 50 μM SA**,** and in the pulse-and-chase experiment (CTR-PC).

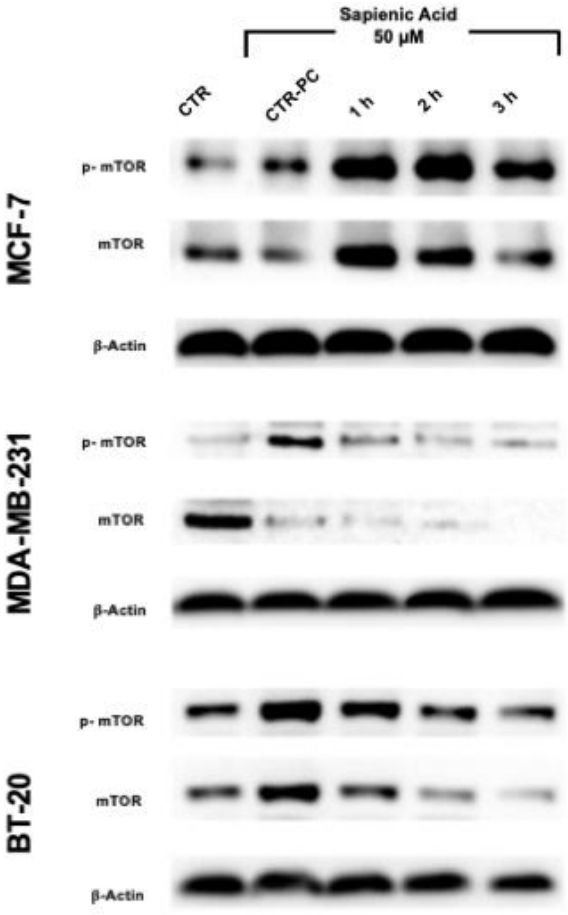

Figure S4. Levels of p-mTOR and mTOR proteins (Western blot) in whole-cell lysates isolated from the breast cancer cell lines, using β-actin as loading controls. 30 µg of protein were loaded for both whole-cell lysate. Detections carried out at 0 (CTR), 1, 2, 3 hours of incubation at 50 µM SA, and in the pulse-and-chase experiment (CTR-PC).

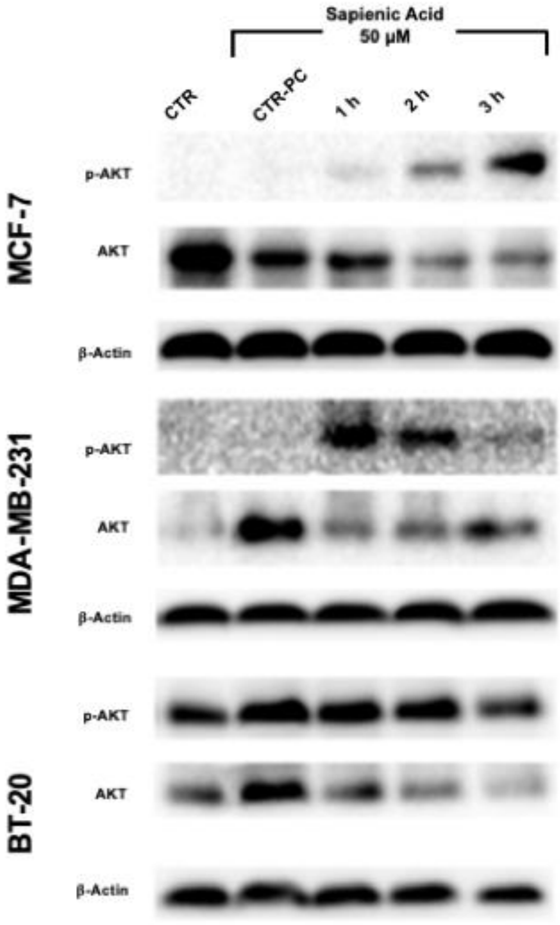

Figure S5. Levels of p-AKT and AKT proteins (Western blot) in whole-cell lysates isolated from the breast cancer cell lines, using β-actin as loading controls. 30 μg of protein were loaded for both whole-cell lysate. Detections carried out at 0 (CTR), 1, 2, 3 hours of incubation at 50 μM SA**,** and in the pulse-and-chase experiment (CTR-PC).
